# Exploring genome-wide – dietary heme iron intake interactions and the risk of type 2 diabetes

**DOI:** 10.3389/fgene.2013.00007

**Published:** 2013-01-30

**Authors:** Louis R. Pasquale, Stephanie J. Loomis, Hugues Aschard, Jae H. Kang, Marilyn C. Cornelis, Lu Qi, Peter Kraft, Frank B. Hu

**Affiliations:** ^1^ Department of Medicine, Channing Division of Network Medicine, Brigham and Women’s Hospital, Harvard Medical SchoolBoston, MA, USA; ^2^Glaucoma Service, Massachusetts Eye and Ear InfirmaryBoston, MA, USA; ^3^Department of Epidemiology, Harvard School of Public HealthBoston, MA, USA; ^4^Department of Nutrition, Harvard School of Public HealthBoston, MA, USA

**Keywords:** type 2 diabetes, gene environment interactions, dietary heme iron, pathway analysis

## Abstract

**Aims/hypothesis:** Genome-wide association studies have identified over 50 new genetic loci for type 2 diabetes (T2D). Several studies conclude that higher dietary heme iron intake increases the risk of T2D. Therefore we assessed whether the relation between genetic loci and T2D is modified by dietary heme iron intake.

**Methods:** We used Affymetrix Genome-Wide Human 6.0 array data [681,770 single nucleotide polymorphisms (SNPs)] and dietary information collected in the Health Professionals Follow-up Study (*n* = 725 cases; *n* = 1,273 controls) and the Nurses’ Health Study (*n* = 1,081 cases; *n* = 1,692 controls). We assessed whether genome-wide SNPs or iron metabolism SNPs interacted with dietary heme iron intake in relation to T2D, testing for associations in each cohort separately and then meta-analyzing to pool the results. Finally, we created 1,000 synthetic pathways matched to an iron metabolism pathway on number of genes, and number of SNPs in each gene. We compared the iron metabolic pathway SNPs with these synthetic SNP assemblies in their relation to T2D to assess if the pathway as a whole interacts with dietary heme iron intake.

**Results:** Using a genomic approach, we found no significant gene–environment interactions with dietary heme iron intake in relation to T2D at a Bonferroni corrected genome-wide significance level of 7.33 ×10^-^^8^ (top SNP in pooled analysis: intergenic rs10980508; *p* = 1.03 × 10^-^^6^). Furthermore, no SNP in the iron metabolic pathway significantly interacted with dietary heme iron intake at a Bonferroni corrected significance level of 2.10 × 10^-^^4^ (top SNP in pooled analysis: rs1805313; *p* = 1.14 × 10^-^^3^). Finally, neither the main genetic effects (pooled empirical *p* by SNP = 0.41), nor gene – dietary heme–iron interactions (pooled empirical *p*-value for the interactions = 0.72) were significant for the iron metabolic pathway as a whole.

**Conclusions:** We found no significant interactions between dietary heme iron intake and common SNPs in relation to T2D.

## INTRODUCTION

Type 2 diabetes (T2D) is a multifactorial condition whereby insulin resistance and beta-cell dysfunction produce glucose metabolism alterations, most notably hyperglycemia, resulting in microvascular and macrovascular complications. T2D affects over 25 million individuals (greater than 8% of the U.S. adult population; American Diabetes Association, 2011) Discovery of the combination of genetic and environmental factors contributing to T2D is essential so that more targeted preventive and management strategies can be devised.

Dietary heme iron intake is the iron derived from hemoglobin, the protein in the red blood cells found in animal foods such as red meat, fish, and poultry. A recent meta-analysis demonstrated that non-heme iron intake (such as iron derived from vegetables), total iron (iron from heme and non-heme sources) or iron supplements were not associated with increased risk of T2D (Bao et al., 2012). However, epidemiologic studies indicate that increased total body iron stores are associated with an increased risk of T2D (Salonen et al., 1998; Jiang et al., 2004b). While the relation between dietary heme iron intake and obesity has not been well-studied, diets with higher heme iron intake have consistently been associated with increased risk of T2D (Jiang et al., 2004a; Lee et al., 2004; Song et al., 2004; Rajpathak et al., 2006). Iron is a catalyst in the formation of hydroxyl radicals, which are powerful pro-oxidants that attack cellular membrane lipids, proteins, and nucleic acids (Nelson, 1999). While heme iron intake is not directly linked to obesity, excess iron stores produced by dietary behaviors may render pancreatic beta cells particularly vulnerable to oxidative stress because they possess a weak antioxidative stress system (Tiedge et al., 1997).

While dietary heme iron intake plays a role in T2D, genome-wide association studies have revealed approximately 50 genetic loci for T2D (Visscher et al., 2012), raising the question of whether gene environment interactions focused on dietary heme iron intake exist in T2D.

In fact, a previous candidate gene association study demonstrated a significant interaction between dietary iron intake and the H63D or C282Y risk variants for *HFE, *the gene for hereditary hemochromatosis (HH), in relation to T2D risk (Qi et al., 2005). HH is a condition where secondary diabetes mellitus is a well-known complication that results from high iron stores in the pancreas (Utzschneider and Kowdley, 2010). In this work, we assessed whether the relation between dietary heme iron intake and T2D is modified by genome-wide single nucleotide polymorphisms (SNPs) or iron metabolic pathway SNPs.

## MATERIALS AND METHODS

### STUDY POPULATION

This study included participants from two longitudinal cohort studies, the Nurses’ Health Study (NHS) and the Health Professionals Follow-up Study (HPFS; Barton et al., 1980; Rimm et al., 1990). The HPFS and the NHS are two populations of men and women, respectively, for whom stored blood, DNA samples, and dietary heme iron intake data are available. The NHS began in 1976 with 121,700 female registered nurses aged 30–55, and the HPFS started in 1986 with 51,529 male health professionals aged 40–75. Both cohorts have been followed-up biennially through mailed questionnaires that gather data on new diseases, diet, and other lifestyle factors.

### ASCERTAINMENT OF TYPE 2 DIABETES:

For participants with a self-report of diabetes on biennial questionnaires, we mailed supplemental questionnaires inquiring about the diagnosis and treatment of their condition, as well as a history of ketoacidosis to corroborate the self-report and to differentiate between type 1 diabetes mellitus and T2D. We applied criteria established by the National Diabetes Data Group until 1997(National Diabetes Data Group, 1979), and then used the revised criteria of the American Diabetes Association from 1998 onward to these supplemental questionnaires (Report of the expert committee on the diagnosis classification of diabetes mellitus, 1997). This approach was found to be valid for identifying T2D as medical record review confirmed 98% of cases in a subsample (Manson et al., 1991).

### FORMATION OF DOMESTIC CASE–CONTROL GROUPS AND GENOTYPING

We accrued 2,591 cases with T2D and 3,508 controls with completed high throughput genotyping from 1986 to 2006 in HPFS and 1980 to 2006 in NHS. From these cases we chose participants of European descent with dietary questionnaire responses prior to diagnosis of T2D. Controls were not strictly matched to cases because some subjects did not consent to have their genotyping data posted on dbGap. Thus we chose controls with complete dietary data that were age matched to cases. Ultimately, 1,806 cases and 2,965 controls were eligible for the study (see **Table 1**). Informed consent was obtained from all participants in this study and the institutional review board at the Harvard School of Public Health approved the study.

**Table 1:** Characteristics of Health Professionals Follow-up Study (HPFS) and Nurses’ Health Study (NHS) cohorts.

Cohort	*N*	Mean heme, SD (mg/day)	Mean body mass index, SD (kg/m^**2**^)	Mean age at baseline, SD (years)
HPFS Cases Controls	1998 725 1273	1.29 (0.43) 1.39 (0.43) 1.23 (0.42)	25.05 (3.46) 27.87 (3.82) 25.01 (2.74)	54.19 (8.36) 52.93 (8.16) 54.91 (8.39)
NHS Cases Controls	2773 1081 1692	1.36 (0.45) 1.41 (0.44) 1.33 (0.45)	26.08 (5.00) 28.66 (4.95) 24.43 (4.29)	47.47 (6.76) 47.31 (6.73) 47.57 (6.78)

*SD, standard deviation.*

We completed genotyping on the Affymetrix Genome-Wide Human 6.0 array at the Broad Institute (Cambridge, MA, USA). Details regarding the quality control measures employed in genotyping these samples have been previously published (Qi et al., 2010). After data cleaning, 706,034 SNPs remained for analysis on 725 cases and 1,273 controls in HPFS. In NHS, a total of 704,409 SNPs remained after data cleaning and were analyzed on 1,081 cases and 1,692 controls.

### DATA ANALYSIS

To assess the interaction between dietary heme iron intake and gene variants in relation to T2D, we created the following logistic regression models:

(1) T2D = β_0_ + β_1_ (age) + β_2_ (body mass index, BMI) + β_3_ (heme) – *Environmental model*
(2) T2D = β_0_ + β_1_ (SNP) + β_2_ (age) + β_3_ (BMI) + β_4_(heme) – *Genetic model*
(3) T2D = β_0_ + β_1_ (SNP) + β_2_ (age) + β_3_ (BMI) + β_4_ (heme) + β_5_ (SNP × heme) – *one-degree of freedom gene–environment (GxE) interaction model*

Except for Model 1 (which we generated in SAS version 9.3, Cary, NC, USA), we formulated these models in each cohort separately using PLINK. Heme iron intake (mg/day), BMI (kg/m^2^, as of 1986 for HPFS and 1980 for NHS) and age (years, as of 1986 for HPFS and 1980 for NHS) were treated as continuous variables. We transformed heme to be centered on the mean value for each cohort. SNPs were coded as 0, 1, or 2 minor alleles. We controlled for the top three eigenvectors in NHS and the top four eigenvectors in HPFS. We also tested models that treated the dietary heme iron intake term as a dichotomous variable divided at the median value in controls.

To test for interactions between dietary heme intake and SNP genotypes in relation to T2D, we utilized a one degree of freedom (1-df) test, which is represented as β_5_ of Model 3. Models 1 and 2 tested the marginal effects of dietary heme iron intake and SNPs respectively, adjusting for age and BMI. We also performed a two degree of freedom joint test (2-df) by comparing the fit of the null model containing the environment exposure only (Model 1) to the model with gene and gene–environment covariates (Model 3) as an alternate test of the gene–environment interaction (Cornelis et al., 2011; Manning et al., 2011). After running these models separately in NHS and HPFS, we performed tests for heterogeneity of the cohort specific results to check for appropriateness of pooling the data. We conducted an inverse variance-weighted fixed-effects meta-analysis of estimates from the two cohorts using the METAL software.^1^ Only SNPs with genotypes available in both cohorts were included in the meta-analysis (*N* = 681,770).

Next we limited our gene–environment interaction analyses to SNPs in the iron metabolic pathway created using the KEGG database ^2^ and other sources (Michal, 1999; Andrews and Schmidt, 2007; Andrews, 2008; Iron Health Alliance, 2009; William, 2009). We identified 237 SNPs in genes coding for enzymes in this pathway that were present on the Affymetrix 6.0 platform (**Table 2**). We repeated the logistic regression analysis of Models 2 and 3 using the iron metabolic pathway SNPs. We used a Bonferroni correction based on the number of SNPs analyzed to establish statistical significance in genome-wide (*p* = 0.05/681,770 = 7.33 × 10^-8^) and pathway analyses (*p* = 0.05/237 = 2.10 × 10^-4^). These estimates of the correction for multiple comparisons are somewhat liberal in that they do not account for the secondary analyses we performed.

**Table 2:** Genes and single nucleotide polymorphisms (SNPs) in the heme iron metabolic pathway on the Affymetrix 6.0 array that passed quality control.

Gene	Chromosome	SNPs
*ACO1*	24	rs1028932, rs10435797, rs10813816, rs13293491, rs10813818, rs2026739, rs7032871, rs10738885, rs16918276, rs3780473, rs3780474, rs4495514, rs13302577, rs7866419, rs7022554, rs4879583, rs10970961, rs7033149, rs10970972, rs10970974, rs7019520, rs13292540, rs13293491, rs3780474
*ALAD*	7	rs8177800, rs8177804, rs818684, rs2761016, rs8177812, rs1805313, rs1805316
*ALAS1*	6	rs352166, rs352167, rs352169, rs11712164, rs352170, rs9813468
*BLVRA*	8	rs10234057, rs849161, rs699510, rs849162, rs849165, rs10233867, rs10268054, rs1317916
*BLVRB*	1	rs2613843
*CP*	17	rs17787768, rs3816893, rs772908, rs16861598, rs16861634, rs16861590, rs4974389, rs701755, rs7652826, rs13072552, rs16861577, rs13075921, rs9853335, rs1879169, rs773050, rs16861579, rs11924961
*CPOX*	2	rs3804622, rs1675534
*FECH*	20	rs1788002, rs1790619, rs492274, rs317806, rs8094527, rs533952, rs12969847, rs1041951, rs7243988, rs317807, rs12454808, rs2269221, rs2269222, rs8099511, rs1736439, rs8092783, rs2272783, rs8095390, rs12968109, rs7242288
*FTH1*	2	rs5904861, rs195154
*HAMP*	1	rs8101606
*HFE*	3	rs1800562, rs2071303, rs2858996
*HFE2*	2	rs10218795, rs7540883
*HMBS*	7	rs1799993, rs1784304, rs1006195, rs494048, rs1144041, rs17075, rs549893
*HMOX1*	7	rs9306300, rs8140669, rs2071749, rs8140370, rs2285112, rs5995097, rs2269533
*HMOX2*	16	rs4786500, rs2160567, rs1362626, rs9302781, rs11076834, rs3789038, rs4786501, rs2270366, rs10500325, rs8063084, rs4785969, rs9929475, rs7192051, rs9936357, rs8048958, rs8055559
*IL6*	7	rs2069837, rs2066992, rs2069835, rs1548216, rs2069842, rs2069840, rs1474347
*IL6R*	16	rs11265618, rs4845626, rs4537545, rs10752641, rs4240872, rs11265610, rs6694817, rs6427658, rs4845618, rs10159236, rs6689393, rs12060250, rs4129267, rs8192282, rs4537545, rs10752641
*IREB2*	18	rs13180, rs2568491, rs9920411, rs924840, rs17483929, rs16969899, rs2656071, rs16969858, rs17483721, rs905742, rs8043227, rs10519198, rs7181486, rs2568483, rs2656073, rs8041628, rs11636431, rs2568492
*SLC11A2*	6	rs17125212, rs2269683, rs224572, rs224573, rs224568, rs224589
*SLC25A37*	33	rs7826247, rs2872716, rs17089392, rs2942194, rs17089394, rs7833754, rs7834536, rs17089335, rs11778179, rs2928672, rs752778, rs7846025, rs3736032, rs7834883, rs7830129, rs2928665, rs7816824, rs10503725, rs17698981, rs2942204, rs2978471, rs2978475, rs17089332, rs11781222, rs7015818, rs12548753, rs7829094, rs13266950, rs17089358, rs2004644, rs4871881, rs4871880, rs7834536
*SLC40A1*	10	rs4667287, rs2304704, rs1439816, rs930373, rs1123110, rs3792079, rs11568350, rs13431938, rs1123109, rs13404407
*SMAD4*	5	rs3764465, rs10502913, rs16952790, rs12457540, rs948588
*STAT3*	16	rs3809758, rs2306580, rs4796646, rs17880900, rs8069645, rs3785898, rs7215104, rs744166, rs4796644, rs8078731, rs8074524, rs6503698, rs1026916, rs7211777, rs3816769, rs9912773
*STEAP3*	30	rs838075, rs708675, rs3769659, rs838074, rs12711924, rs41527945, rs7596511, rs838083, rs708672, rs838072, rs708670, rs865688, rs838095, rs12465926, rs11888609, rs838103, rs12990907, rs10182241, rs838090, rs1867856, rs10188946, rs838092, rs4592854, rs12104548, rs838079, rs6542517, rs838102, rs1867749, rs13401854, rs6720040
*TF*	37	rs4854759, rs8177201, rs1358022, rs8177203, rs8177306, rs1049296, rs2715627, rs1799852, rs7638018, rs4241357, rs7633232, rs6778321, rs1800277, rs8177241, rs8177232, rs2715631, rs8177277, rs8177235, rs8177220, rs8177238, rs8177233, rs2715632, rs8177272, rs12493168, rs2718796, rs1115219, rs8177253, rs8177318, rs8177224, rs7628133, rs8177248, rs1525892, rs8177262, rs7645538, rs8177191, rs8177215, rs3811657
*TFR2*	1	rs4521695
*TFRC*	6	rs3933, rs3804141, rs3804142, rs4927866, rs480760, rs12330245
*UROD*	1	rs13948
*UROS*	12	rs10510149, rs1571278, rs2281956, rs10794025, rs3814624, rs11244653, rs10901450, rs2027515, rs3740179, rs10751533, rs2149019, rs12251135

To evaluate whether the iron metabolic pathway as a whole might interact with dietary heme intake, we assessed whether the iron metabolic pathway SNP panel was enriched with variants strongly associated with T2D. We compared the distribution of *p*-values in the SNPs from the iron metabolic pathway with the distribution of SNPs from 1,000 permuted “synthetic pathways” generated by randomly picking SNPs available on the platform that were not in the iron metabolic pathway. To ensure an adequate comparison, all synthetic pathways were constructed such that they had the same number of genes and the same number of SNPs per gene ±10% as the heme pathway. Enrichment of SNPs with low *p*-values was evaluated by comparing *C*_obs_, the count of SNPs with a *p*-value below a given significance threshold *T* in the heme metabolic pathway, to *C*_syn_, the corresponding count derived in the synthetic pathways. The empirical *p*-value (for both the main genetic effect and the gene–environment interaction term) related to enrichment for a given *T* was derived as the number of times *C*_obs_ was higher than *C*_syn_ divided by 1,000, the total number of synthetic pathways.

## RESULTS

Type 2 diabetes cases were similar in age compared to controls in men (overall mean ± SD = 54.3 ± 8.4 years) and women (47.5 ± 6.8 years). As expected, cases had higher BMI and higher mean dietary heme iron intake than controls in men and women (**Table 1**). Dietary heme iron intake was adversely associated with T2D [OR = 1.36 (1.17, 1.58); pooled *p* = 7.51 × 10^-5^; Model 1]. As expected, the top SNP associated with T2D was in *TCF7L2 *(rs7901695; pooled *p*-value =1.88 ××10^-14^) (Model 2).

Using the 1-df test (Model 3), no gene–environment interaction achieved genome-wide significance level. The most significant interaction with continuous dietary heme iron intake was rs10980508 (pooled *p* = 1.03 × 10^-6^; an intergenic SNP between muscle, skeletal, receptor tyrosine kinase (*MUSK)* and Sushi, von Willebrand factor type A, EGF, and pentraxin domains-containing 1 (*SVEP1*; **Table 3**). The 2-df test revealed that SNPs in *TCF7L2* had genome-wide margin association with T2D but did not reveal new marginal gene effects of genome-wide significance; nor was there significant interaction between *TCF7L2* and dietary heme iron intake in T2D (data not shown). When we generated models substituting dietary heme intake with red meat, processed meat, and total meat, we found similar results with top marginal genetic effects in *TCF7L2*; yet, Model 3 did not yield significant gene–environment interactions (data not shown).

**Table 3:** Top 10 *p*-values from genome-wide SNP–heme interactions predicting type 2 diabetes adjusting for age, body mass index, dietary heme iron intake, and eigenvectors using a one degree of freedom test from meta-analysis of the Health Professionals Follow-up Study and Nurses’ Health Study.^a^

Chromosome	SNP	Beta, HPFS^b^	Beta, NHS^b^	*p*-Value, HPFS^b^	*p*-Value, NHS^b^	*p*-Value, pooled^b,c^	Associated gene
9	rs10980508	0.96	0.64	1.81 × 10^-4^	1.23 × 10^-3^	1.03 × 10^-6^	Upstream of *MUSK*, downstream of *SVEP1*
9	rs12378245	0.96	0.61	1.77 × 10^-4^	1.76 × 10^-3^	1.51 × 10^-6^	Upstream of *MUSK*, downstream of *SVEP1*
9	rs10817049	0.92	0.61	3.09 × 10^-4^	1.61 × 10^-3^	2.16 × 10^-6^	Upstream of *MUSK*, downstream of *SVEP1*
9	rs7048110	0.96	0.58	1.80 × 10^-4^	2.75 × 10^-3^	2.53 × 10^-6^	Upstream of *MUSK*, downstream of *SVEP1*
9	rs10817052	0.90	0.60	4.43 × 10^-4^	1.93 × 10^-3^	3.53 × 10^-6^	Upstream of *MUSK*, downstream of *SVEP1*
16	rs17177078	1.48	0.83	1.50 × 10^-4^	5.66 × 10^-3^	5.06 × 10^-6^	Intron in *TNRC6A*
9	rs10980495	0.91	0.57	3.60 × 10^-4^	3.33 × 10^-3^	5.49 × 10^-6^	Upstream of *MUSK*, downstream of *SVEP1*
7	rs1525739	-0.58	-0.44	7.85 × 10^-4^	2.11 × 10^-3^	6.26 × 10^-6^	Downstream of *AGR3*, upstream of *AGR2*
9	rs10448267	0.94	0.54	2.58 × 10^-4^	4.95 × 10^-3^	6.58 × 10^-6^	Upstream of *MUSK*, downstream of *SVEP1*
22	rs470089	-0.59	-0.55	2.90 × 10^-3^	9.43 × 10^-4^	8.64 × 10^-6^	Intron in *SULT4A1*

a Model controls for BMI (kg/m^2^) and age (years), both as continuous variables, continuous mg dietary heme intake per day, centered on the mean heme value for each cohort and eigenvectors 1–3 for NHS or eigenvectors 1–4 for HPFS. SNPs are coded as 0, 1, or 2 minor alleles.

b Betas and *p*-values are from the β_5_ (gene variant × dietary heme iron intake) term in Model 3: T2D = β_1_ (BMI) + β_2_ (age) + β_3_ (dietary heme iron intake) + β_4_ (gene variant) + β_5_ (gene variant × dietary heme iron intake) + eigenvectors.

c *P* for heterogeneity is >0.05 (lowest *p*-value is 0.4).HPFS, Health Professionals Follow-up Study; NHS, Nurses’ Health Study; MUSK, muscle, skeletal, receptor tyrosine kinase; SVEP1, Sushi, von Willebrand factor type A, EGF, and pentraxin domains-containing 1; *TNRC6A*, trinucleotide repeat-containing gene 6A; *AGR3*, anterior gradient 3; AGR2, anterior gradient 2; SULT4A1, sulfotransferase family 4A, member 1.

No significant iron metabolism SNP – dietary heme iron intake interaction was detected with the 1-df test (Model 3) in relation to T2D (top SNP rs1805313; in *ALAD* (delta-aminolevulinate dehydratase); pooled *p* = 1.14 × 10^-3^; Bonferroni corrected significance level *p* = 2.10 × 10^-4^). The 2-df test of gene and gene–environment interactions also did not reveal any significant interactions between dietary heme iron intake and SNPs in the iron metabolic pathway SNP in pooled analyses (data not shown).

Compared with synthetic pathways, the iron metabolic pathway was not associated with T2D when we performed the analyses by SNP (pooled empirical *p* by SNP = 0.41). Similar null results were obtained when interactions with dietary heme iron intake were considered (pooled empirical *p*-value for the interactions =0.72). Interactions between various forms of dietary meat intake and the iron metabolic pathway were also not significant.

## DISCUSSION

Neither the 1-df test nor the 2-df test revealed any genome-wide significant interactions between dietary heme iron intake and genomic SNPs in T2D. Furthermore, the relation between an iron metabolic pathway SNP panel and T2D was not modified by dietary heme iron intake. Finally the iron metabolic pathway was not enriched with SNPs related to T2D.

There could be several possible reasons for the null results reported here. First, despite its large size (*n* = 4,771) our study could be underpowered to find modest interaction terms. In fact, only in a log additive model would we achieve 80% power to detect a genome-wide environmental interaction effect of 1.8 (assumes minor allele frequency = 0.4; genetic relative risk = 1.2, and relative risk of the highest tertile of dietary heme iron intake = 1.3). Nonetheless we had ~80% power to discover an interaction effect of 1.5 between iron metabolic SNPs and dietary heme iron intake in relation to T2D using similar assumptions in a dominant inheritance model. Second, power could be compromised due to inherent error in measuring dietary heme iron intake. Third, self-reported iron intake, while collected using a validated food frequency questionnaire, could be prone to recall bias. Finally, the reason why a higher dietary heme iron dietary intake increases the risk of T2D could be solely related to environmental influences (Lee et al., 2004).

A prior study, using a candidate approach, did find a marginal interaction between *HFE *and dietary heme iron intake in T2D (*p* = 0.03; Qi et al., 2005). While we did not discover any new gene-dietary heme iron interactions, more studies using serum biomarkers as surrogates of dietary heme iron intake might point to new gene–iron intake interactions in T2D.

## Conflict of Interest Statement:

The authors declare that the research was conducted in the absence of any commercial or financial relationships that could be construed as a potential conflict of interest.

## Footnotes

1 www.sph.umich.edu/csg/abecasis/metal/

2 www.genome.jp/kegg/pathway.html

## Abbreviations

1-df test: one degree of freedom gene–environment interaction test
2-df test: two degree of freedom gene–environment interaction test
HH: hereditary hemochromatosis
HPFS: Health Professionals Follow-Up Study
NHS: Nurses’ Health Study
QC: quality control
SNP: single nucleotide polymorphism
T2D: type 2 diabetes.
